# A randomized single-blind non-inferiority trial of delayed start with drospirenone-only and ethinyl estradiol-gestodene pills for ovulation inhibition

**DOI:** 10.1038/s41598-024-64753-7

**Published:** 2024-06-19

**Authors:** Atist Ratanasaengsuang, Sutira Uaamnuichai, Somsook Santibenchakul, Rachanee Wongwathanavikrom, Sukanya Chaikittisilpa, Natkrita Pohthipornthawat, Charoen Taweepolcharoen, Unnop Jaisamrarn, Phanupong Phutrakool

**Affiliations:** 1 Department of Obstetrics and Gynecology, Faculty of Medicine, Chulalongkorn University, Bangkok, Thailand; 2 Department of Obstetrics and Gynecology, King Chulalongkorn Memorial Hospital, Bangkok, Thailand; 3 Center of Excellence in Menopause and Aging Women Health, Department of Obstetrics and Gynecology, Faculty of Medicine, Chulalongkorn University, Bangkok, Thailand; 4 Chula Data Management Center, Faculty of Medicine, Chulalongkorn University, Bangkok, Thailand; 5 Center of Excellence in Preventive & Integrative Medicine, Faculty of Medicine, Chulalongkorn University, Bangkok, Thailand

**Keywords:** Randomized controlled trials, Endocrinology

## Abstract

We compared the efficacy of 4 mg drospirenone (DRSP) progestin-only pills (POPs) versus combined oral contraceptive pills (COCs) containing 0.02 mg of ethinyl estradiol (EE) and 0.075 mg of gestodene (GS) in ovulation inhibition and inducing unfavorable cervical mucus changes using a delayed-starting approach. This randomized controlled trial involved 36 participants aged 18–45 years. The major outcomes included ovulation inhibition assessed using the Hoogland and Skouby score, and cervical mucus permeability, assessed using the modified World Health Organization score. The results demonstrated ovulation inhibition rates of 77.8% for the EE/GS group and 88.9% for the DRSP group. The risk ratio and absolute risk reduction were 0.50 (95% confidence interval [CI]: 0.10, 2.40) and − 0.11 (95% CI: − 0.35, 0.13), respectively, satisfying the 20% non-inferiority margin threshold. The median time to achieve unfavorable cervical mucus changes was comparable between the DRSP (3 days, interquartile range [IQR]: 6 days) and EE/GS (3.5 days, IQR: 4 days) groups. However, the DRSP group had a higher incidence of unscheduled vaginal bleeding (55.56% vs. 11.11%; p = 0.005). DRSP-only pills, initiated on days 7–9 of the menstrual cycle, were non-inferior to EE/GS pills in ovulation inhibition. However, they exhibited delayed unfavorable cervical mucus changes compared to the standard two-day backup recommendation.

Clinical trial registration: Thai Clinical Trials Registry (TCTR20220819001) https://www.thaiclinicaltrials.org/show/TCTR20220819001.

## Introduction

Oral contraceptive pills (OCPs) are the second-most commonly used contraceptives worldwide^1^. In Thailand, OCPs are the preferred contraceptive method^2^. Two types of OCPs are available: combined oral contraceptive pills (COCs) containing both estrogen (ethinyl estradiol [EE]) and progestin, and progestin-only pills (POPs). COCs primarily inhibit ovulation by suppressing luteinizing hormone (LH) through the progestin component^3^, which also affects cervical mucus formation, fallopian tube function, and endometrial decidualization^4–6^. The estrogen component in COCs minimizes unscheduled bleeding and enhances progestin action by increasing progesterone receptors and suppressing follicle-stimulating hormones (FSH), yielding better efficacy than POPs^3,7–9^. In contrast, POPs primarily alter cervical mucus and have a lower ovulation-inhibition rate than COCs, with their endometrial effects potentially aiding in pregnancy prevention^6,10^. Despite showing greater contraceptive efficacy and less unscheduled bleeding than POPs, COCs are contraindicated in women with certain medical conditions, whereas the contraindications to POPs are limited^9^. Newer POPs containing 4 mg of drospirenone (DRSP) have shown superior ovulation inhibition (96.3%) than desogestrel-based POPs, but are contraindicated in individuals with renal insufficiency or untreated hyperaldosteronism^11^. DRSP-based POPs showed a low overall ovulation rate (0.9%) despite multiple intentional 24-h delays in pill intake, representing the same safety window of delayed intake as COCs^12^.

Access to contraception can be delayed if a woman needs to wait to initiate the OCPs at the beginning of the menstrual cycle. Quick-starting contraception, which is initiated immediately at the woman’s request, may lead to fewer unintended pregnancies due to increased access, convenience, and better adherence and continuation^13–15^. International guidelines recommend quick-starting with backup contraception^9,16–19^. In this approach, while 7 days of additional contraception are recommended for COCs based on their mechanism of ovulation inhibition, only 2 days of additional contraception with POPs are recommended considering cervical mucus blockage, the primary mechanism of action of traditional POPs^9,16^. Despite these recommendations, few clinical studies have demonstrated the efficacy of OCPs in delayed-starting cycles, particularly for ovulation inhibition and follicular activity^15,20–26^.

We compared 4 mg DRSP-only pills with ultra-low-dose COCs for ovulation inhibition, starting treatment on days 7–9 of the menstrual cycle. This timing reflects common delays in real-world contraceptive use, and the choice of ultra-low-dose COCs aligns with current trends in reducing hormone levels while maintaining effectiveness, and is supported by recent studies on delayed COCs initiation^20,21,27,28^. We set a 20% non-inferiority margin in ovulation inhibition compared to the active control, based on existing evidence^20^. Additionally, we evaluated the onset of changes in cervical mucus permeability, crucial for POPs recommendations.

## Methods

### Study design

We conducted a single-blinded (investigator-blinded), parallel-group, randomized, controlled, non-inferiority trial. The protocol was registered in the Thai Clinical Trials Registry (TCTR) and approved on August 19, 2022 (TCTR20220819001). The research protocol was designed in accordance with the International Good Clinical Practice regulations and the Declaration of Helsinki and was approved by the Institutional Review Board (IRB) of the Faculty of Medicine, Chulalongkorn University (0243/65). All participants provided written informed consent before enrollment, and received 500 bath (approximately $15) for each visit as compensation for the time spent on the project.

Randomization was performed using a computer-generated sequence by a biostatistician who was not involved in data analysis. The allocation ratio between the experimental treatment and active control was 1:1 with a block of four randomizations. Participants were assigned to groups using individually sealed envelopes, which were opened by a research assistant not involved in data analysis. Medication for each patient was provided in unique envelopes. To ensure investigator blinding in a single-blind study, participants were instructed to keep the medication in these envelopes during clinic visits. The primary investigator (RA), who was blinded to the study allocation and participants’ hormone levels, performed transvaginal ultrasonography (TVUS) and cervical mucus evaluations. The investigators involved in data analysis (RA, US, PP, and SS) were blinded to the study intervention.

### Participants

The participants were recruited from the Family Planning and Reproductive Health Unit of King Chulalongkorn Memorial Hospital (KCMH), Thailand and Internet advertisements^29,30^. We included women aged 18–45 years (body mass index [BMI], 18–30 kg/m^2^) with a history of regular menstrual cycles of 24–38 days. The exclusion criteria were contraindications to estrogen or progestin therapy^9^, pregnancy or lactation, exogenous hormone use (estrogen, progestin, or testosterone) in the past 3 months, or the presence of precancerous or cancerous cervical lesions, ovarian cysts, suspected ovarian tumors, or a pre-existing dominant follicle larger than 10 mm on the initial screening TVUS, which is crucial for accurately determining the participant’s menstrual date.

### Study protocol

The initial screening was conducted on the first or second day of the menstrual cycle (visit 0), during which a complete medical history, general physical examination, and TVUS were performed. Unless male or female sterilization was used for contraception, the participants were instructed to use male condoms during the study period.

The first visit after enrollment (visit 1, also referred to as the starting medication day) was timed to occur on days 7–9 of the menstrual cycle. During this visit, TVUS was used to assess ovarian activity, cervical mucus was collected, and blood was drawn for the measurement of serum estradiol, progesterone, and LH levels. At this visit, participants were randomized into two groups, receiving either DRSP or EE/gestodene (GS), and were provided with their respective medications and a menstrual diary for recording.

Follow-up visits were initially scheduled every 2–3 days to monitor for signs of ovulation inhibition, evaluate cervical mucus quality, and measure serum levels of estradiol, progesterone, and LH. These visits continued at this frequency until ovulation was observed or until cervical mucus assessments yielded a modified WHO score of ≤ 4 or the largest follicle diameter (LFD) was < 13 mm on two consecutive occasions, indicating unfavorable fertility conditions^31^. Following these outcomes, the frequency of the follow-up visits was adjusted to a weekly basis, allowing for continued assessments via TVUS until the completion of the 28-day medication course.

### Study medication

We used a DRSP-only pill (Slinda; Laboratorios Leon Farma S.A., Spain) containing 4 mg of DRSP per tablet. This medication is also marketed in the United States of America (USA) and Europe as Slynd. One packet of the study medication contained 24 active tablets and four placebo tablets. The control medication was a COC pill containing 0.02 mg of EE and 0.075 mg of GS (Annylyn 28; Thai Nakorn Patana, Thailand). This drug is available in the USA and Europe as Meliane, Sunya, Femodette, and Millinette 20/75. Each packet included 21 active tablets and seven placebo tablets. The ultralow dose EE/GS was chosen as a control due to its proven efficacy in delay starting approaches from a prior study and its widespread use in Thailand^20^. The participants were instructed to take the study medication at night before bed, and note the time of tablet ingestion, bleeding, and bothersome adverse effects in their paper diaries. Bleeding was defined as uterine bleeding, which in the view of the participant, required use of sanitary protection^32^. If participants missed a pill, they were instructed to take it as soon as possible. Medication reminders were sent via chat every day at 9.00 p.m., and the participants were asked to respond by reaffirming medication intake. Despite being considered to have deviated from the protocol, participants who missed a pill for more than 24 h were still included in the intention to treat analysis.

### Outcomes

The primary outcome was ovulation inhibition, as defined by the Hoogland and Skouby score, as shown in Fig. S1^20,33^. Secondary outcomes were the time required to achieve unfavorable mucus after treatment initiation and adverse events. Follicle like structures refers to the follicles or cystic ovarian structures observed on TVUS, which were measured by averaging the two largest perpendicular diameters in millimeters (mm). TVUS was performed using a Phillips Affiniti 70G (SN: US919F1869) with a transvaginal probe (C10-3V; 10–3 MHz).

Morning blood samples were collected in 5-mL clot-activated tubes and sent within 30 min to the laboratory center, Faculty of Medicine, Chulalongkorn University, ISO 15189. An electrochemiluminescence immunoassay (ECLIA, Cobas, Switzerland) was used to measure serum estradiol and progesterone levels using the competition method and to measure serum LH using the sandwich method. The intra- and inter-assay coefficients of variation were less than 4%. The minimum detection limits were 18.35 pmol/L for estradiol, 0.159 nmol/L for progesterone, and 0.1 IU/L for LH. Our study used the standard progesterone level of 5 nmol/L or 1.57 ng/mL, as defined by the Hoogland and Skouby score, to indicate ovulation^33^.

#### Follicular dynamics

After completing the treatment cycle, follicular dynamics were sorted into four categories based on the Hoogland and Skouby score, as shown in Fig. S2^20^.

#### Cervical mucus assessment

Cotton swabs were used to wipe the external cervical os. A syringe was then inserted 1–2 cm into the cervical canal, and gentle suction was applied to aspirate the mucus. Within 30 min, a trained scientist (WR) assessed the mucus using the methodology in WHO Laboratory Manual 5th Edition, as shown in Fig. S3^31^. Each parameter was photographed, recorded, and confirmed by the principal investigator (RA). Inconclusive results were discussed by the RA and WR.

We used a modified WHO scoring system with a maximum score of 12, and defined scores ≤ 4 as indicating unfavorable mucus, as shown in Fig. S4^31^. Since progesterone reduces mucus permeability, cycles in which ovulation occurred before the cervical mucus became unfavorable were excluded from the analysis.

#### Bothersome adverse events

Participants recorded the adverse events in their diaries. Unscheduled bleeding was defined by evidence of blood loss that requires the use of sanitary protection with a tampon, pad or pantyliner during active tablet ingestion^32^. The diary logs were evaluated at each follow-up appointment.

### Statistical methods

#### Sample size

Duijkers et al. stated that 4 mg of DRSP taken on day 1–2 of menstruation is highly effective in inhibiting ovulation, even with a 24-h delay protocol^11,12^. Jirakittidul et al. reported that ultra-low dose COCs containing EE/GS had a 95.6% ovulation-inhibition rate in the delayed-starting approach^20^. We aimed to determine if DRSP 4 mg inhibited ovulation comparable to that of EE/GS. Our study used a non-inferiority design with 80% power, a 1-sided test alpha of 5%, and a non-inferiority margin of 20%. The final sample size was 18 per group, accounting for a 30% dropout rate.

#### Statistical analysis

Research Electronic Data Capture (REDCap), administered by Chulalongkorn University, was used to collect and manage the data^34^. STATA version 17 (StataCorp. 2021. Stata Statistical Software: Release 17. College Station, TX: StataCorp LLC.) was used for analyses. Descriptive statistics were used as appropriate. To evaluate the intergroup differences in the ovulation-inhibition, we used univariable logistic regression and presented the results as risk ratios (RRs) and 95% confidence intervals (CIs). We calculated the absolute risk difference (ADR) and 95% CI to show that our results were within the 20% non-inferiority margin. The multivariable analyses were adjusted for age. The cumulative incidence of ovulation was analyzed using the Kaplan–Meier survival estimation and the log-rank test for survival function equality. Statistical significance was set at p < 0.05. We conducted both per protocol (PP) and intention-to-treat (ITT) analyses but only reported the ITT results if both revealed comparable results. If there were differences, both results would be reported. For the ITT analysis, we imputed data using worst-case scenarios for most outcomes, including ovulation, follicular dynamics, and cervical mucus permeability. We imputed the data on the mean LFD, estradiol, and LH levels throughout the treatment cycle using the mean variable of that study group.

## Results

**Figure 1:**
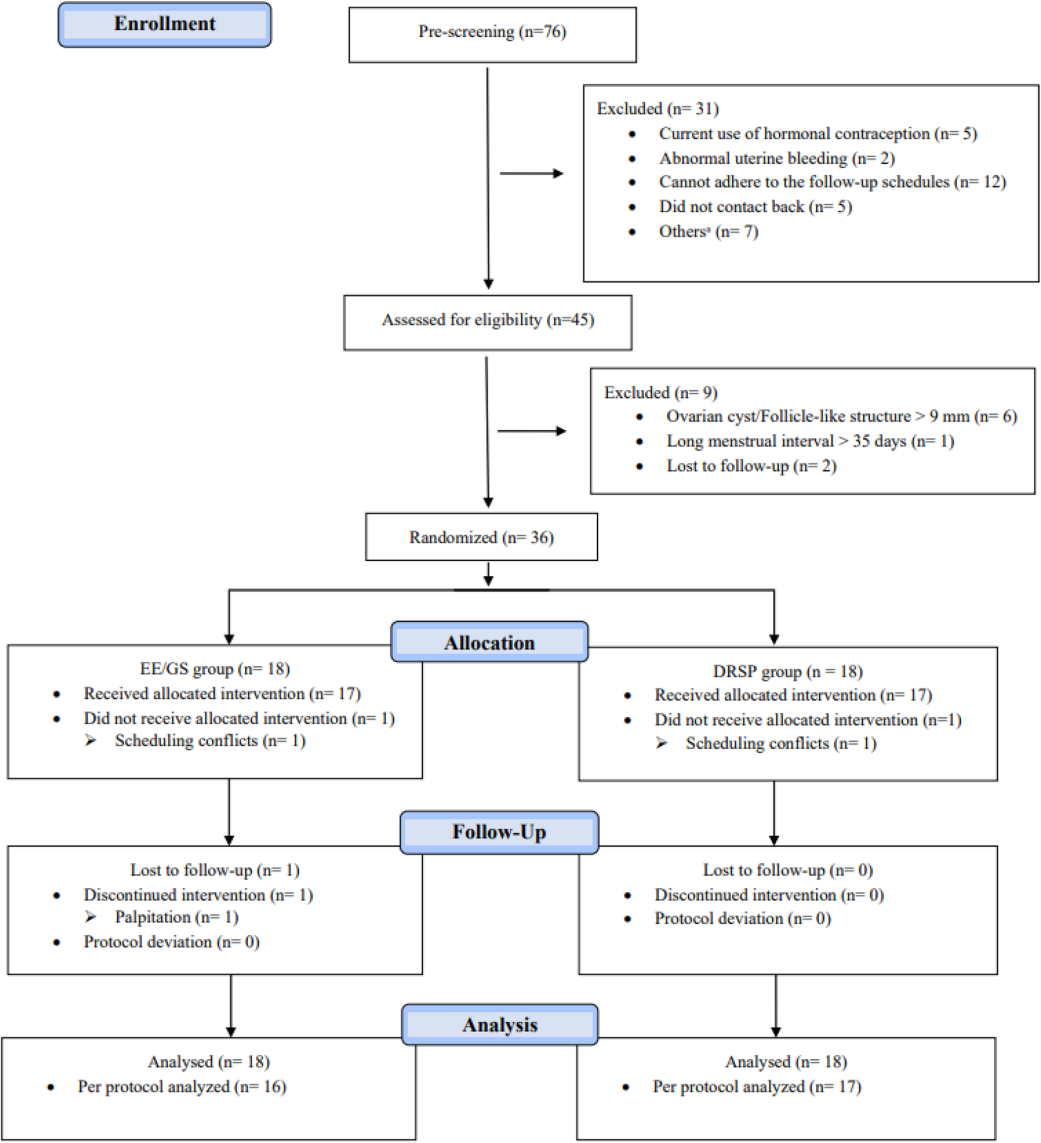
Consort flow chart. *EE/GS* ethinyl estradiol 0.02 mg plus gestodene 0.075 mg, *DRSP* 4 mg drospirenone. ^a^Age older than 45 (n = 2), Wrong contact address (n = 4), BMI over than 30 kg/m^2^ (n = 1).

Between August and December 2022, thirty-six participants were randomized to the control group (EE/GS) or study group (DRSP), as shown in Fig. 1. One participant from each group left the study because of scheduling conflicts; both were randomized, but neither took the study medication. One participant of the EE/GS group took the pill for 4 days and requested withdrawal from the study after experiencing adverse effects. No protocol deviations were observed. Finally, the PP analysis included 16 and 17 participants from the EE/GS and DRSP groups, respectively, while the ITT analysis included all participants.

The two study groups had comparable demographic characteristics (Table 1). The BMI distribution according to WHO and Center for Disease Control and Prevention (CDC) criteria showed that 5.56% and 11.11% of the participants in the EE/GS and DRSP groups, respectively, were clinically obese (BMI ≥ 27.5 and ≥ 30 kg/m^2^, respectively)^35,36^. The proportions of participants who began taking the study medication on days 7, 8, and 9 of the menstruation cycle and the Hoogland scores of 1, 2, and 4, respectively, were comparable between the groups (Table 2). LFD on the starting medication day did not differ significantly between the two groups, as shown in Table S1.

**Table 1:** Baseline characteristics.

Basic characteristics	EE/GS n = 18	DRSP n = 18	p-value^a^
Age			
Mean ± SD	36.68 ± 7.72	33.05 ± 6.85	–
Median (IQR)	39.78 (30.33–43.32)	32.89 (30.34–37.27)	0.076
Age group, n (%)			0.182
≤ 35 years	7 (38.89)	11 (61.11)	
> 35–45 years	11 (61.11)	7 (38.89)	
Body mass index (kg/m^2^)^35^, n (%)			0.097
Underweight (< 18.5)	1 (5.56)	2 (11.11)	
Normal (18.5–< 23)	5 (27.78)	10 (55.56)	
Overweight (23.0–< 27.5)	11 (61.11)	4 (22.22)	
Obese (≥ 27.5)	1 (5.56)	2 (11.11)	
Body mass index (kg/m^2^)^36^, n (%)			0.662
Underweight (< 18.5)	1 (5.56)	2 (11.11)	
Normal (18.5–< 25)	10 (55.56)	11 (61.11)	
Overweight (25.0–< 30.0)	6 (33.33)	3 (16.67)	
Obese (≥ 30)	1 (5.56)	2 (11.11)	
Menstrual history			
Interval (days)			
Mean ± SD	29.83 ± 2.75	29.17 ± 2.83	–
Median (IQR)	29.50 (28.00–31.00)	28.50 (27.00–31.00)	0.424
Duration (days)			
Mean ± SD	4.61 ± 1.04	4.83 ± 1.34	0.582
Median (IQR)	5 (4–5)	5 (4–5)	–
Amount (pads/days)			
Mean ± SD	4.06 ± 0.80	3.83 ± 1.15	0.506
Median (IQR)	4 (3–5)	4 (3–4)	–
Menstrual associated symptoms, n (%)			
Pelvic pain	13 (72.22)	14 (77.78)	> 0.999
Nausea	–	1 (5.56)	> 0.999
Diarrhea	–	1 (5.56)	> 0.999
Headache	1 (5.56)	1 (5.56)	> 0.999
Acne	3 (16.67)	1 (5.56)	0.603
Breast tenderness	2 (11.11)	9 (50.00)	0.027
Fatigue	2 (11.11)	4 (22.22)	0.658
Bloating	1 (5.56)	1 (5.56)	> 0.999
Mood swing	–	1 (5.56)	> 0.999
Other^b^	3 (16.67)	3 (16.67)	> 0.999
Recent contraceptive use, n (%)			
Condoms	13 (72.22)	13 (72.22)	> 0.999
Combined oral contraceptive pills	1 (5.56)	–	> 0.999
Withdrawal technique	2 (11.11)	3 (16.67)	> 0.999
Female sterilization	4 (22.22)	–	0.104

*EE/GS* ethinyl estradiol 0.02 mg plus gestodene 0.075 mg, *DRSP* 4 mg drospirenone, *SD* standard deviation, *IQR* interquartile range.

^a^Student *t*-test, Wilcoxon rank-sum test, Chi-square or Fisher’s exact tests as appropriate.

^b^Backache and loss of appetite.

**Table 2:** Characteristic on the starting medication day.

On the starting medication day	EE/GS n = 18	DRSP n = 18	p-value^a^
Cycle day at starting medication, n (%)			0.319
Day 7	6 (35.29)	5 (29.41)	
Day 8	5 (29.41)	9 (52.94)	
Day 9	6 (35.29)	3 (17.65)	
Largest follicle diameter			
Mean ± SD	10.75 ± 3.80	9.76 ± 2.03	0.351
Median (IQR)	11.70 (8.40–11.80)	10.40 (8.40–10.80)	–
Estradiol (pg/mL)			
Mean ± SD	112.62 ± 94.54	68.34 ± 33.91	–
Median (IQR)	66.54 (37.89, 139.19)	69.87 (42.89, 76.24)	0.502
Progesterone (ng/dL)			
Mean ± SD	0.24 ± 0.17	0.39 ± 0.83	–
Median (IQR)	0.19 (0.17, 0.30)	0.13 (0.10, 0.30)	0.407
LH (IU/L)			
Mean ± SD	7.45 ± 4.83	6.52 ± 1.98	–
Median (IQR)	5.55 (4.34–8.20)	6.71 (4.76–7.50)	0.945
Cervical mucus (modified WHO score)			
Mean ± SD	5.35 ± 2.00	4.41 ± 1.42	0.123
Median (IQR)	5 (4–6)	4 (4–5)	–
Hoogland and Skouby Score at starting medication day, n (%)			0.785
No activity	8 (47.06)	9 (52.94)	
Potential activity	6 (35.29)	7 (41.18)	
Non-active FLS	–	–	
Active FLS	3 (17.65)	1 (5.88)	
LUF	–	–	
Ovulation	–	–	

*EE/GS* ethinyl estradiol 0.02 mg plus gestodene 0.075 mg, *DRSP* 4 mg drospirenone, *SD* standard deviation, *IQR* interquartile range, *FLS* follicle like structure, *LUF* luteinized unruptured follicle.

^a^Student *t*-test, Wilcoxon rank-sum test, Chi-square or fisher's exact tests as appropriate.

**Figure 2:**
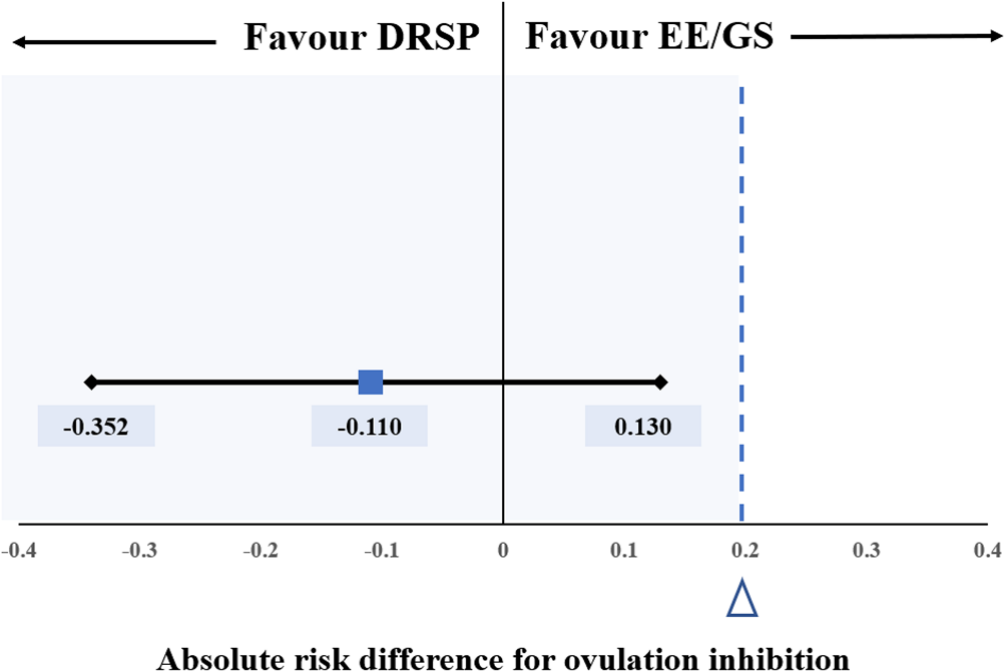
Absolute risk difference for ovulation inhibition. Error bars indicate 2-sided 95% Cis. The dashed blue line at x = ∆ represents the 20% noninferiority margin. The region shaded blue to the left of x = ∆ represents the zone of noninferiority. Absolute risk difference − 0.11 (− 0.352, 0.130) declared DRSP is noninferior. *EE/GS* ethinyl estradiol 0.02 mg plus gestodene 0.075 mg, *DRSP* 4 mg drospirenone.

**Figure 3:**
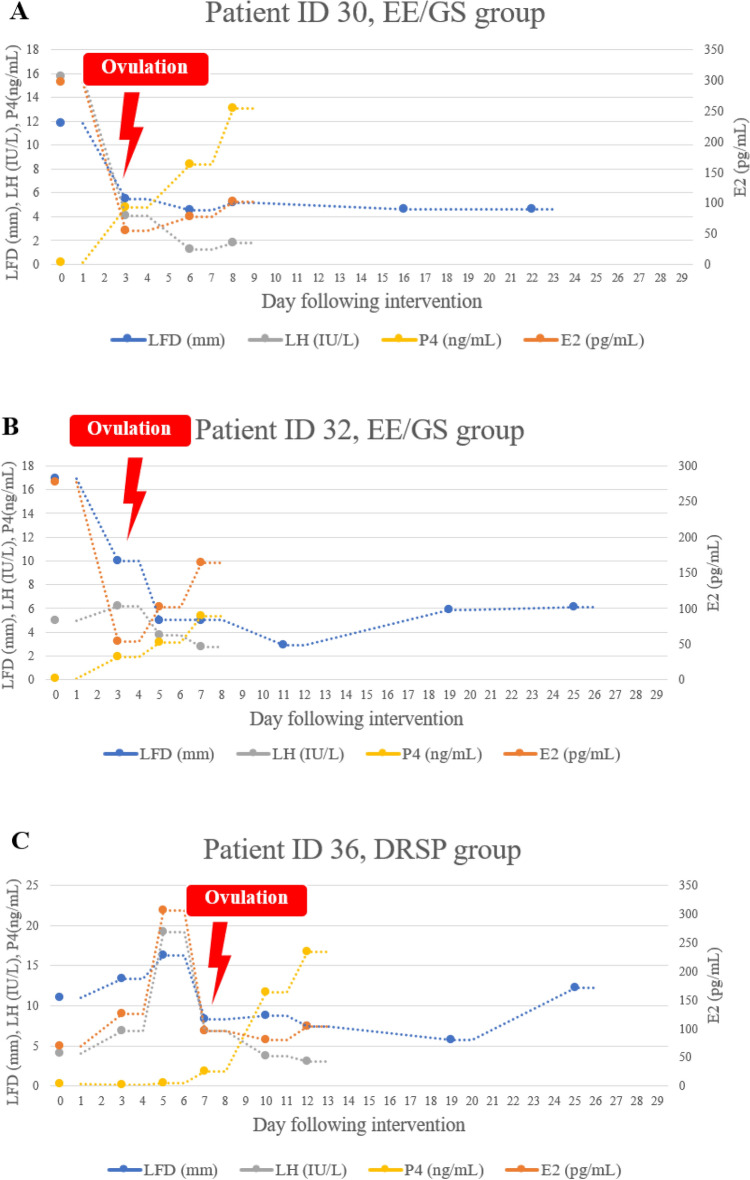
Follicular activity and hormonal levels among the three participants who ovulated. (**A**) Patient ID 30 in the EE/GS group started medication on day seven of menstruation when the LFD was 11.8 mm, the LH level was 15.72 IU/mL, and the estradiol level was 296.67 ng/mL. Ovulation was suspected on day three after the intervention, as TVUS showed the sudden disappearance of LFD. On day eight, progesterone level rose to 13.10 ng/mL. (**B**) Patient ID 32 in the EE/GS group started medication on day eight of menstruation when the LFD was 16.9 mm, the LH level was 4.94 IU/mL, and the estradiol level was 277.87 ng/mL. Ovulation was suspected on day three after the intervention, as TVUS showed collapsed LFD. On day seven, progesterone level rose to 5.36 ng/mL. (**C**) Patient ID 36 in the DRSP group started medication on day eight of menstruation when the LFD was 11.0 mm, the LH was 4.09 IU/mL, and the estradiol level was 69.87 ng/mL. On day five following intervention, LFD was 16.3 mm, the LH level was 19.9 IU/L, and the estradiol level was 305.64 ng/mL. Ovulation was suspected on day seven after the intervention, as TVUS showed collapsed LFD. On day 12, progesterone level rose to 16.71 ng/mL. *EE/GS* ethinyl estradiol 0.020 mg plus gestodene 0.075 mg, *DRSP* 4 mg drospirenone, *Day 0* starting medication day, *LFD* leading follicle diameter, *LH* Luteinizing hormone, *P4* Progesterone, *E2* Estradiol, *TVUS* transvaginal ultrasound.

In both EE/GS and DRSP groups, ultrasound findings indicated suspected follicular rupture in up to one-third of cases (35.71% in EE/GS and 25.0% in DRSP). Despite these findings, there was no significant increase in progesterone levels, which remained below 5 nmol/L (1.57 ng/mL) during at least two visits (spanning approximately 4–6 days) following the suspected follicular rupture in these participants. Ovulation inhibition, defined by follicular dynamics based on the Hoogland and Skouby score^33^, occurred in four (77.8%) and two (88.9%) patients in the EE/GS and DRSP groups, respectively (RR = 0.50, 95% CI: 0.104, 2.395, p = 0.386; Table 3). The absolute risk difference was − 0.110 (95% CI: − 0.352, 0.130), of which the 20% non-inferiority margin was 0.2 (Fig. 2). The multivariable analysis adjusted for age showed comparable results. In Fig. 3, we present the PP analysis of LFD, hormone levels, and ovulation dates of the three participants who ovulated. Two participants in the EE/GS group ovulated 3 days after beginning the study medication, whereas one participant in the DRSP group ovulated 7 days after starting the medication.

**Table 3:** Results.

	EE/GS n = 18	DRSP n = 18	Risk ratio	p-value^a^
Follicular dynamics based on Hoogland and Skouby Score, n (%)				
No follicular growth	5 (27.78)	3 (16.67)	0.60 (0.17, 2.14)	0.432
Regression	7 (38.89)	3 (16.67)	0.43 (0.13, 1.40)	0.161
Persisting follicular cyst	2 (11.11)	10 (55.56)	5.00 (1.27, 19.69)	0.021
Ovulation	4 (22.22)	2 (11.11)	0.50 (0.10, 2.40)	0.386
Hoogland and Skouby Score at the ending of treatment cycle, n (%)				
No activity	12 (66.67)	4 (22.22)	–	0.018
Potential activity	2 (11.11)	3 (16.67)	–	1.0
Non-active FLS	0 (0)	1 (5.56)	–	1.0
Active FLS	4 (22.22)	10 (55.56)	–	0.086
LUF (5)	0	0	–	–
Ovulation (6)	0	0	–	–
Duration of archiving modified WHO score ≤ 4 from starting medication day				
Mean ± SD	7.39 ± 10.20	4.28 ± 6.97	–	0.293
Median (IQR)	3.50 (4)	3 (6)	–	–

*EE/GS* ethinyl estradiol 0.02 mg plus gestodene 0.075 mg, *DRSP* 4 mg drospirenone, *SD* standard deviation, *IQR* interquartile range.

^a^Student *t*-test, Wilcoxon rank-sum test, Chi-square or Fisher’s exact tests as appropriate.

**Figure 4:**
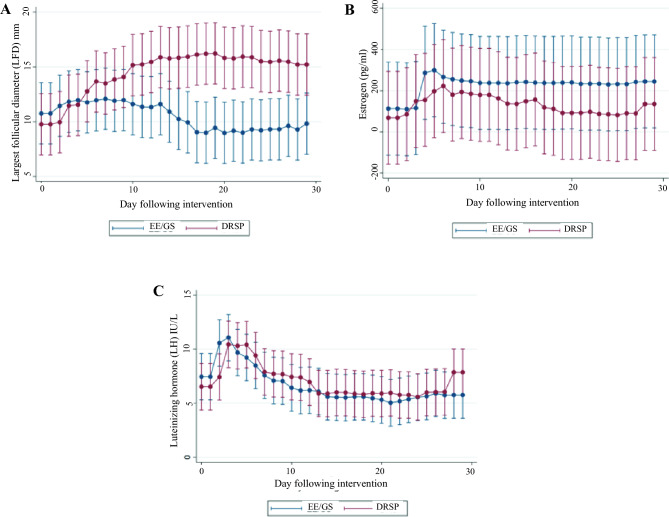
Characteristics of treatment cycle. (**A**) Largest follicle diameter (LFD), (**B**) Estrogen level (pg/mL) and (**C**) LH level (IU/L). *EE/GS* ethinyl estradiol 0.02 mg plus gestodene 0.075 mg, *DRSP* 4 mg drospirenone.

**Figure 5:**
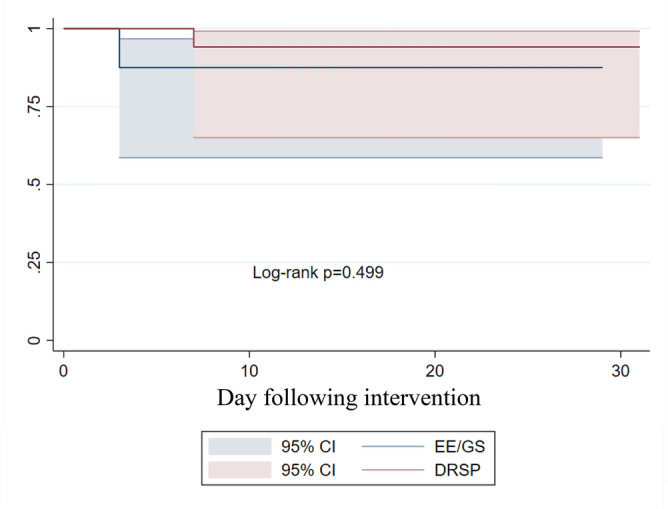
Kaplan–Meier survival curve of intention to treat population illustrates cumulative incidence of ovulation inhibition of delayed-starting on day 7–9. *EE/GS* ethinyl estradiol 0.02 mg plus gestodene 0.075 mg, *DRSP* 4 mg drospirenone.

Figure 4 illustrates the LFD, estrogen, and LH levels throughout the study period. As the cycles progressed, LFD tended to decrease in the EE/GS group, while the serum estrogen levels tended to be lower in the DRSP group. The LH levels were comparable between the two groups. During the study, 36 participants were observed for 861 person-days and three instances of ovulation occurred, as determined by the PP analysis. The Kaplan–Meier curve showed an incidence of 3.48 per 1000 person-days (95% CI: 1.12–10.80), with no significant difference between the two groups (p = 0.499), as shown in Fig. 5. Table 3 presents the Hoogland and Skouby scores at the end of the treatment cycle, in which approximately half of the participants in the EE/GS group showed no ovarian activity.

The mean duration for achieving the modified WHO cervical mucus score ≤ 4 after taking the medication is shown in Table 3. It took a median time (IQR) of 3.50 (4) in the EE/GS and 3 (6) days in the DRSP group to achieve unfavorable cervical mucus change. Within 7 days, cervical mucus changes were observed in nearly all participants, with one exception from each group. In the DRSP group, one participant did not experience cervical mucus changes. In contrast, the exception in the EE/GS group experienced these changes on 10 days after starting the study medication.

The incidence of unscheduled bleeding was significantly higher in the DRSP group (55.56%) than in the EE/GS group (10%; p = 0.005), as shown in Table 4. One participant in the EE/GS group discontinued the study due to palpitation after 4 days of medicine administration, when physical examination and electrocardiogram showed normal findings and normal sinus rhythm, respectively. Other adverse events were comparable between the groups (Table 4). The blood pressure and weight measurements obtained before and after medication administration are shown in Table S2.

**Table 4:** Bothersome adverse events.

Adverse Events	EE/GS n = 18 n (%)	DRSP n = 18 n (%)	p-value^a^
Unscheduled bleeding^b^	2 (11.11)	10 (55.56)	0.005
Duration^c^: Mean ± SD	3.5 ± 3.54	6.5 ± 3.78	
Duration^c^: Median (IQR)	3.5 (5)	5.5 (7)	0.387^d^
Nausea	7 (38.89)	2 (11.11)	0.121
Pelvic pain	7 (38.89)	4 (22.22)	0.471
Headache	5 (27.78)	2 (11.11)	0.402
Acne	4 (22.22)	6 (33.33)	0.711
Breast tenderness	2 (11.11)	6 (33.33)	0.228
Palpitation	1 (5.55)	0 (0)	> 0.999

*EE/GS* ethinyl estradiol 0.02 mg plus gestodene 0.075 mg, *DRSP* 4 mg drospirenone.

^a^Chi-square or Fisher’s exact tests as appropriate.

^b^Number of participants experiencing unscheduled bleeding.

^c^Duration of unscheduled bleeding in individuals experiencing this condition.

^d^Mann–Whitney *U* test.

## Discussion

### Main findings

This study established a 20% non-inferiority margin of ovulation inhibition between ultra-low dose COCs (EE/GS) and POPs containing 4 mg of DRSP in a delayed-starting regimen on days 7–9 of the menstrual cycle, of which ovulation inhibition was demonstrated in 77.8% and 88.9% of participants, respectively. Most participants experienced unfavorable cervical mucus changes within the first 7 days, which exceeded the standard two-day backup recommendation^16^.

Jirakittidul et al.’s study on a delayed-starting regimen of ultralow-dose COCs (EE/GS) in a design with a fixed-cycle day^20^ revealed that the ovulation-inhibition rate was 95.6% when medication was started on days 7–9 of the menstrual cycle. However, while majority of our participants started the intervention on days 8–9, representing only half of those with no demonstrated ovarian activity, the corresponding proportion in the study by Jirakittidul et al. was approximately 75%^20^. This may explain the differences in the ovulation rates in the two studies.

Several studies have reported discrepancies between ultrasound findings and serum progesterone levels when determining the Hoogland and Skouby score^37–39^. Therefore, our primary method for determining ovulation based on the Hoogland and Skouby scores relied on serum progesterone elevation. This discrepancy between the ultrasound findings and progesterone levels posed a challenge in establishing ovulation using the Hoogland and Skouby score. Other studies facing this challenge have defined ovulation based solely on progesterone levels when ultrasound findings were inconclusive and have used anovulation status as the outcome measure^37,39^. Additionally, measuring estradiol, which must exceed 200 pg/mL for at least 50 h to induce LH release preceding ovulation, could offer clearer ovulation detection^40^. However, this approach requires frequent testing, which was beyond the scope of this study but could be considered in future research for enhanced accuracy.

Among the discrepant findings for hormonal and follicular dynamics, one distinct pattern was an increase in LH levels, indicating an LH surge, followed by a decline to baseline levels, despite follicular rupture occurring over time. This pattern deviated from the normal cycle, wherein follicular rupture occurred 28–36 h after the onset of the LH surge^41^. Erden et al.^42^ identified three physiological events occurring after the LH surge in the natural ovulatory cycle: resumption of first meiotic division at a low LH level; luteinization of granulosa cells at higher LH levels; and follicle wall rupture at very high LH levels^43^. Erden et al. concluded that the interval between the onset of the LH surge and ovulation typically ranged from 22 to 56 h in a normal ovulatory cycle^42,44^. Thus, the delayed follicular rupture following an LH surge without an increase in progesterone levels could be attributed to the contraceptive mechanism inducing an abnormal LH surge^43^.

A significant disparity was observed between the two groups, with approximately two-thirds of the participants in the EE/GS group showing no follicular growth or regression and only one-third of the participants in the DRSP group showing this pattern. This observation was further supported by the time trend of the LFD (Fig. 4). Participants in the DRSP group exhibited larger LFD values as the cycles progressed. Notably, a persistent follicle was the most distinctive characteristic of the DRSP group, with approximately half of the participants experiencing this phenomenon^45^. This finding can be attributed to the estrogen component of combined oral contraceptives (COC), which inhibits FSH secretion, suppressing follicle growth and reducing overall follicular activity^3,7,8^. Another explanation could be variations in progestin types, which may warrant further study.

Branche et al. studied the pharmacodynamics of delayed-starting desogestrel 0.075 mg, used after ulipristal acetate emergency contraception^46^. In one arm of their study, POPs were administered alone when the follicle reached 14–16 mm, which corresponds to the late follicular phase. An unfavorable cervical mucus was achieved in 76% (16/21) of the participants within 2 days of medicine administration and in all participants within 4 days. In contrast, our study administered the medicine when the LFD was approximately 10 mm. Within 10 days, all participants, except one in the DRSP group, exhibited a change to unfavorable cervical mucus. Our findings imply that 2 days may be insufficient for backup; however, given the limited scope and sample size of our study, we cannot definitively recommend extending the backup period for DRSP-based POPs. Additional research is needed to explore the timing of achieving cervical mucus change when using pills containing only DRSP, as this may differ from that in other POPs.

Han et al.’s review^47^ criticized the lack of specific studies correlating changes in the cervical mucus score with the risk of pregnancy and highlighted that the recommendation regarding cervical mucus was based on expert opinions with limited clinical evidence. Additionally, the Sentinel study on cervical mucus did not assess the drug’s impact on the periovulatory period, which shows the highest mucus production^10^. Most clinical guidelines over almost half a century have failed to reference the primary literature on the contraceptive effects of cervical secretions^9,16–19^.

### Strengths and limitations

The effectiveness of POPs for delayed-starting contraception has been evaluated in only a few studies^46^. Our study addressed this gap by evaluating the effectiveness of POPs using a Hoogland and Skouby score, the standard algorithm for assessing ovulation^33^. To ensure the validity and reliability of the results and to minimize bias, cervical mucus scoring was performed by a well-trained researcher blinded to the study intervention. Cervical mucus was evaluated according to the WHO Laboratory Manual 5th Edition methodology criteria^31^. We also implemented measures to monitor participants’ adherence. We requested that all participants respond to a chat message regarding medicine ingestion, thereby minimizing protocol violations.

While daily monitoring yields the best assessment of ovulation outcomes, we mitigated this limitation by incorporating progesterone level assessments and serial ultrasound scans conducted by a single operator. Another limitation was that our protocol was designed to evaluate ovulation inhibition, which influenced the focus of our follow-up visits. Thus, the evaluation interval for cervical mucus was set at either 36 h or 60 h after medication administration, potentially affecting the timing of the cervical mucus change and its interpretation. While the randomization process yielded comparable baseline characteristics among the study participants, those in the DRSP group had a smaller LFD on the starting day of medication. Although the difference was small and did not reach statistical significance, it could still contribute to better ovulation suppression. Additionally, since our study was designed based on non-inferiority, we could not determine whether POPs were superior to COCs in terms of ovulation inhibition with a delayed-starting approach. Critically, the choice of a 20% non-inferiority margin, while consistent with prior studies^20,21^, was not derived from specific empirical evidence, which may limit the precision of our conclusions regarding the comparative effectiveness of the contraceptive methods studied. Considering the fixed-cycle study design in clinical practice, addressing the knowledge gap associated with subgroup evaluation based on menstrual cycle duration, particularly in women with short menstrual intervals, is crucial. Moreover, the challenges arising when POPs are administered in the late follicular phase also require consideration.

## Conclusions

Our study demonstrated the non-inferiority of ovulation inhibition by 4-mg DRSP-only pills in comparison with COCs containing EE 0.02 mg and GS 0.075 mg in a delayed-starting approach when medication was administered on days 7–9 of the menstrual cycle. We found delayed unfavorable cervical mucus changes that exceeded the 2-day backup recommendation.

### Supplementary Information

Supplementary Information.

## Data Availability

Data generated and analyzed during the current study are available from the corresponding author, Somsook Santibenchakul, upon reasonable and appropriately justified requests. Interested researchers are encouraged to contact Somsook Santibenchakul directly to discuss data access and collaboration opportunities. All requests will be reviewed for compliance with ethical and confidentiality standards before any data can be shared.
