# Supplementary material for: A randomized single-blind non-inferiority trial of delayed start with drospirenone-only and ethinyl estradiol-gestodene pills for ovulation inhibition
Source: Sci Rep. 2024 Jun 19;14:14151. doi: 10.1038/s41598-024-64753-7 (PMC11187189; doi:10.1038/s41598-024-64753-7)
Supplement: Supplementary file 1 — Supplementary Information. [file 41598_2024_64753_MOESM1_ESM.docx]

| Figure S1 | | | | |
| --- | --- | --- | --- | --- |
| Grade of ovarian activity | | Follicle-like structures (FLS) size (mm) | Hormone | |
|  |  |  | Estradiol (pmol/L)ᵃ | Progesterone (nmol/L)ᵇ |
| 1 | No activity | ≤ 10 |  |  |
| 2 | Potential activity | > 10 |  |  |
| 3 | Non-active follicle like structures (FLS) | > 13 | ≤ 100 |  |
| 4 | Active follicle like structures (FLS) | > 13 | > 100 | ≤ 5 |
| 5 | Luteinized unruptured follicle (LUF) | > 13, persisting | > 100 | > 5 |
| 6 | Ovulation | > 13, ruptured | > 100 | > 5 |
| Figure S1: Hoogland and Skouby score^(23)^  ᵃEstradiol 100 pmol/L = 27.24 pg/ml  ᵇProgesterone 5 nmol/L = 1.57 ng/ml | | | | |

| Figure S2 | | |
| --- | --- | --- |
| 1 | No follicular growth | No follicle development exceeding 13 mm throughout the cycle |
| 2 | Regression | Follicles initially exceeding 13 mm regressed and decreased below 13 mm toward the end of the study period, indicated by a decline from modified Hoogland-Skouby scores of 3-4 |
| 3 | Persisting follicular cysts | A growing follicle exceeding 13 mm persisted beyond the study period and showed a modified Hoogland and Skouby score of 3-4 throughout the cycle |
| 4 | Ovulation | Modified Hoogland and Skouby score increased to 5 or 6 |
| Figure S2: Follicular dynamics based on the Hoogland and Skouby score^(22, 24)^ | | |

Figure S3

**A**


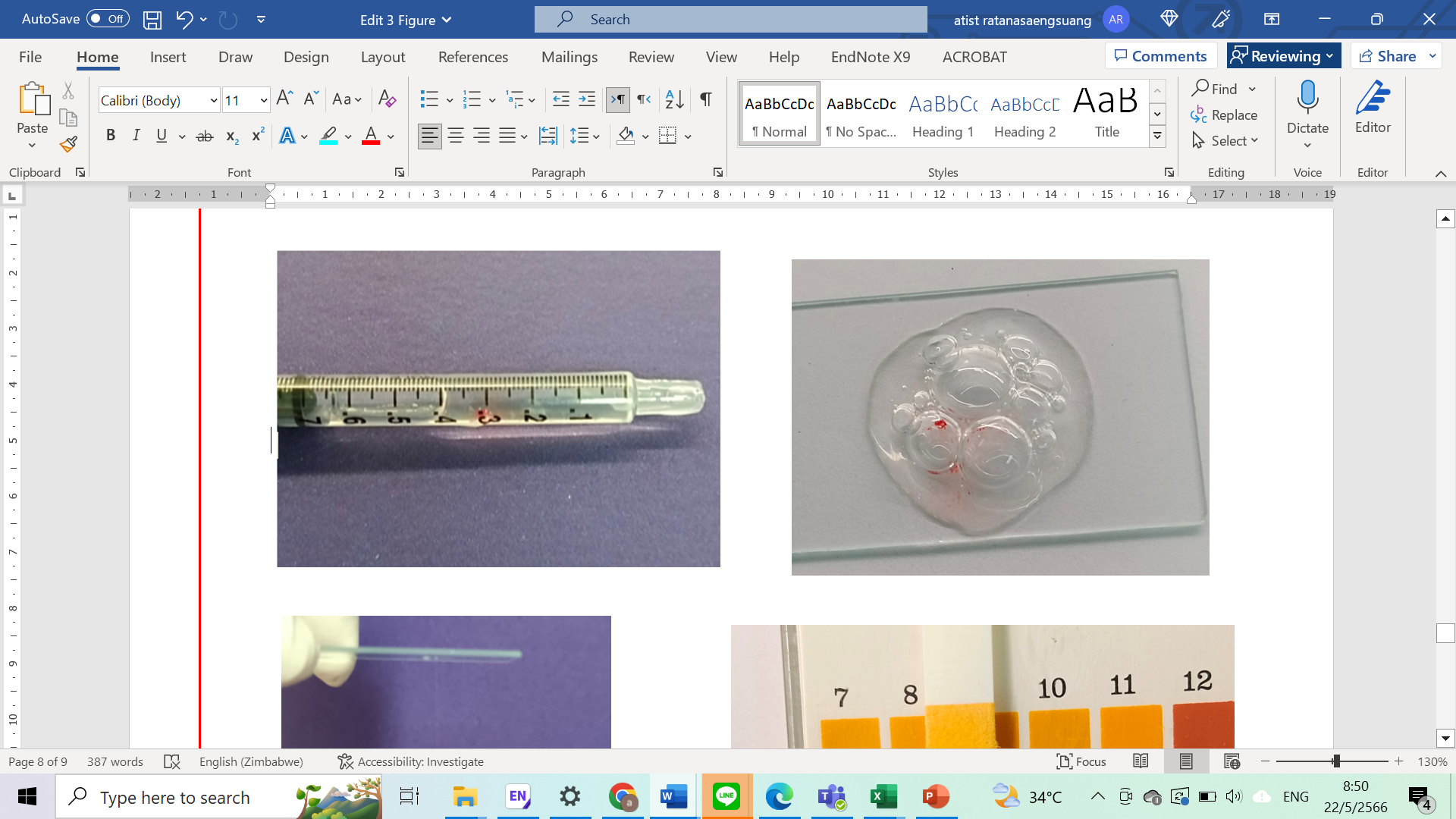

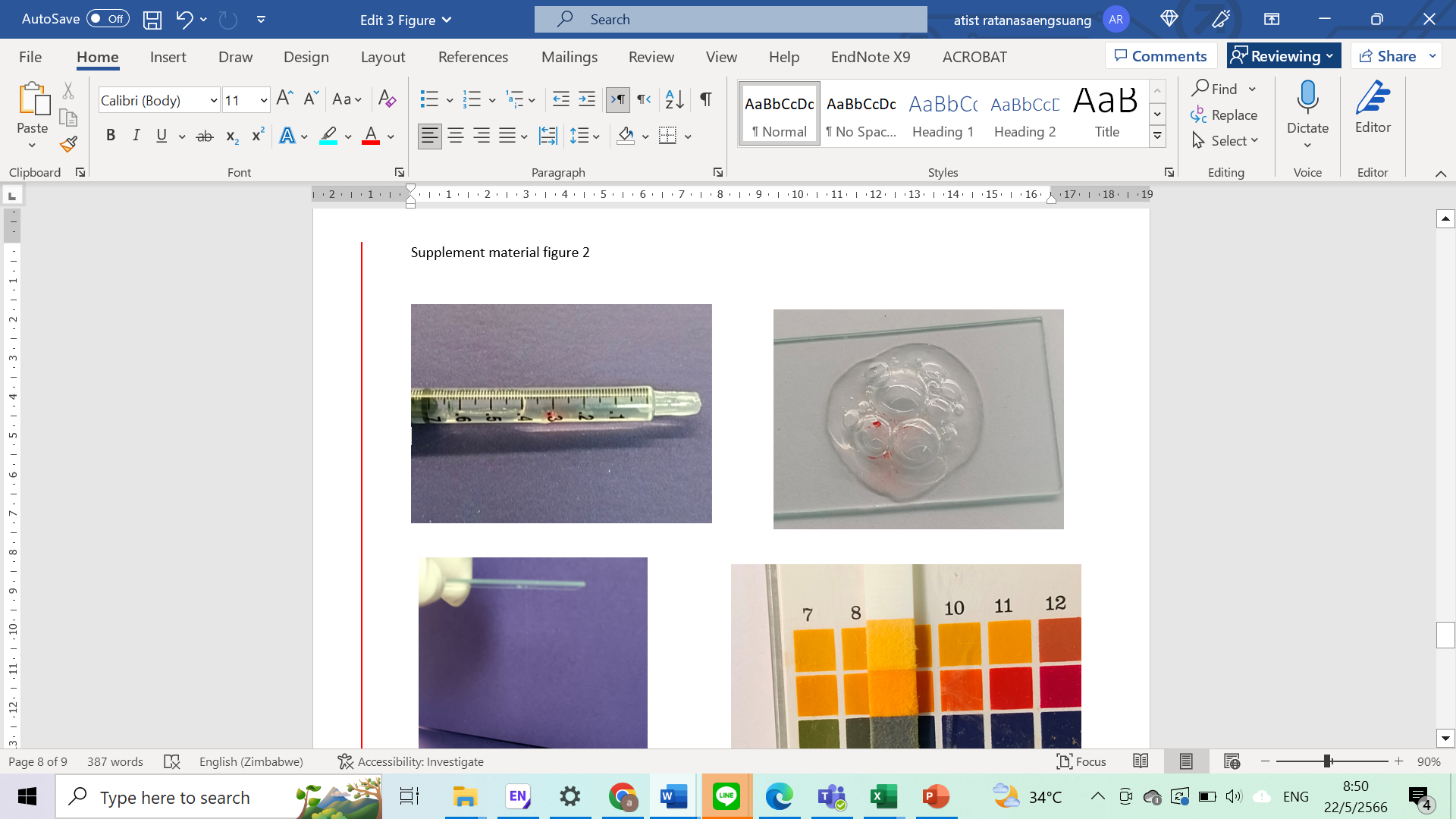


**B**


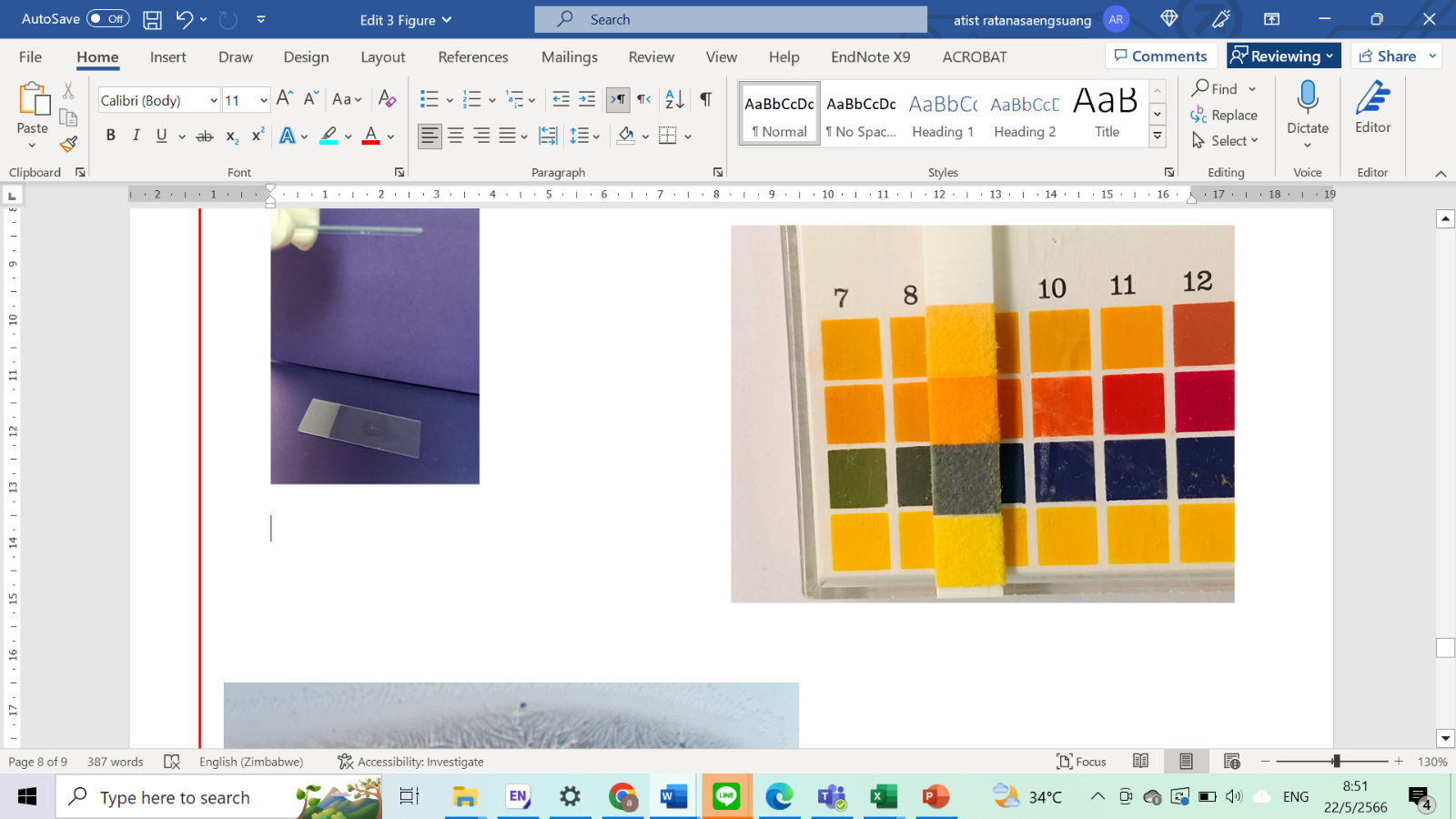

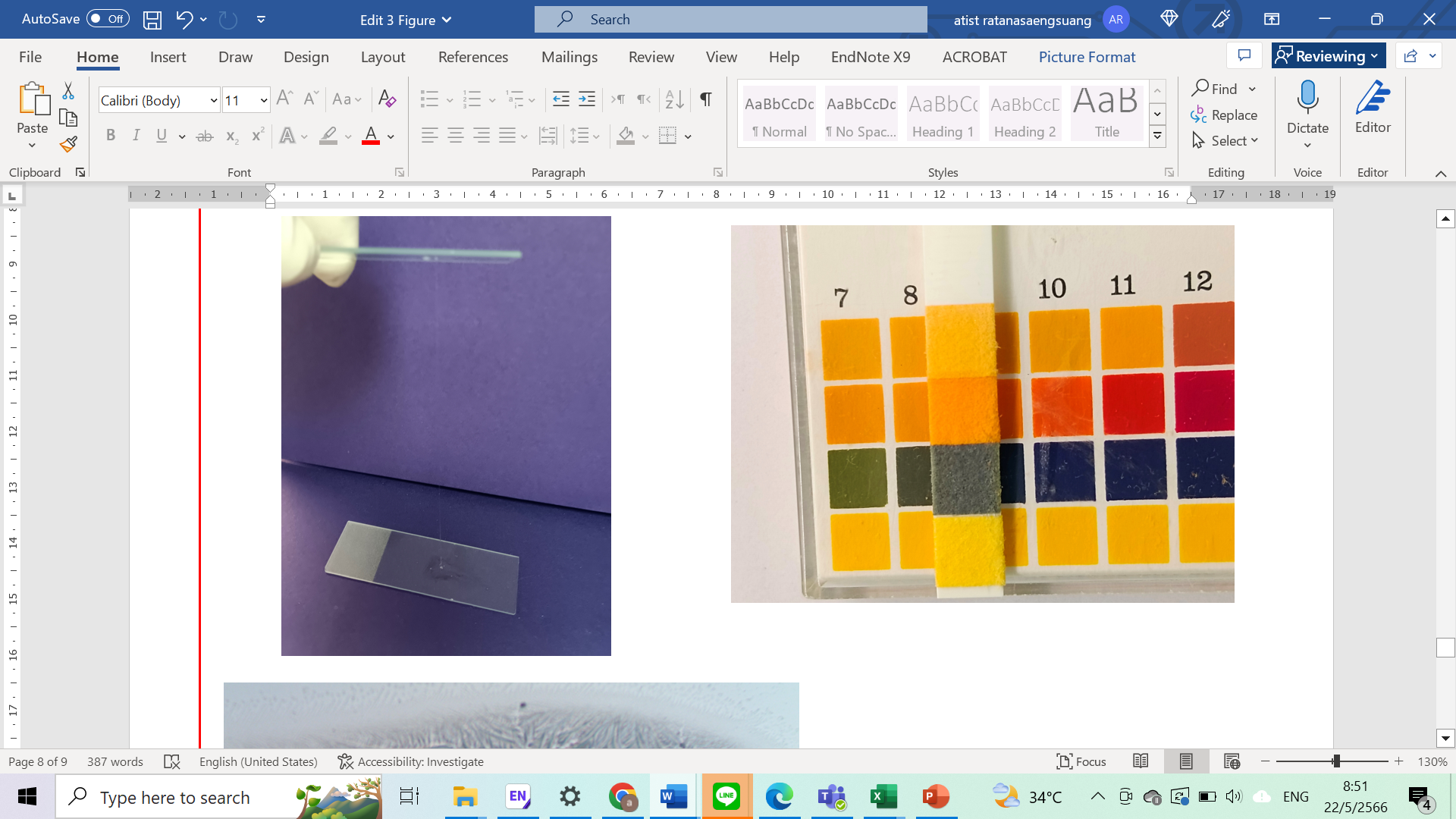


**D**

**C**


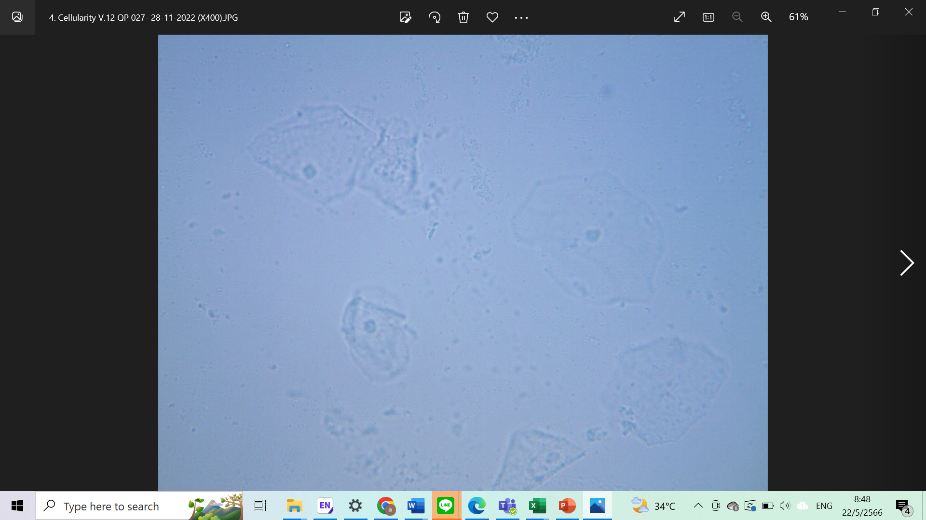

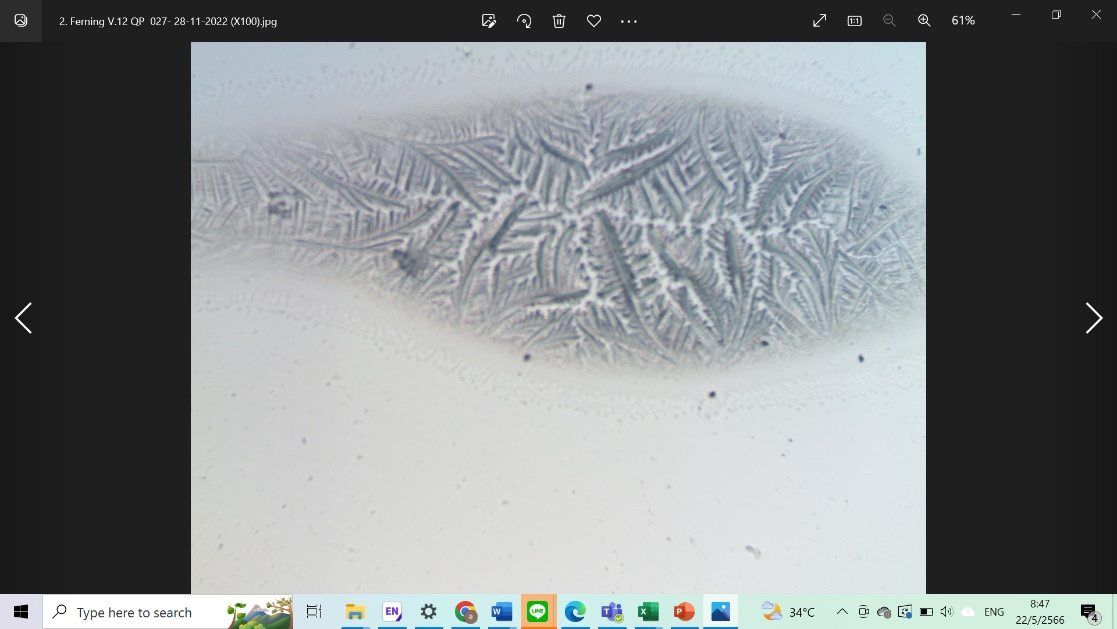


**F**

**E**

Figure S3: Steps of cervical mucus assessment

Example of cervical mucus assessment in Patient ID 27, on day 27 following intervention; (A) Collected cervical mucus in a 1 mL syringe; (B) A drop of cervical mucus on a glass slide demonstrates the watery viscosity indicated by WHO score 3; (C) Spinnbarkeit testing reveals a length of stretched cervical mucus of 10.3 cm, as indicated by WHO score 3; (D) pH testing indicates pH = 8, (E) Ferning reveals tertiary stem ferning indicated by WHO score 3; (F) Cellularity of vaginal epithelium 8 cells per high power field indicates WHO score 2

| Figure S4 | | | | |
| --- | --- | --- | --- | --- |
| Score: | 0 | 1 | 2 | 3 |
| Viscosity | Thick, highly viscous | Intermediate viscosity | Mildly viscous | Watery, minimally viscous |
| Ferning | No crystallization | Atypical fern formation | Primary and secondary stem | Tertiary and quaternary |
| Spinnbarkeit | < 1cm | 1-4 cm | 5-8 cm | 9 cm or more |
| Cellularity | > 20 cells/HPF | 11-20 cells/HPF | 1-10 cells/HPF | 0 cell |
| Figure S4: Modified World Health Organization score for cervical mucus permeability^a(23)^  ^a^A modified WHO scoring system with a maximum score of 12, and defining a scores ≤4 as indicating unfavorable mucus | | | | |

| Table S1 | | | |
| --- | --- | --- | --- |
| **LFD on the starting medication day** | **EE/GS (n = 18)**  **n (%)** | **DRSP (n = 18)**  **n (%)** | **p-value^a^** |
| < 10 mm | 8 (44.44) | 9 (50) | 1.0 |
| 10-13 mm | 6 (33.33) | 7 (38.89) | 1.0 |
| > 13 mm | 3 (16.67) | 1 (5.56) | 0.6 |
| Table S1: Largest follicle diameter on the day of starting study medication  Abbreviations: EE/GS, ethinyl estradiol 0.02 mg plus gestodene 0.075 mg; DRSP, 4 mg drospirenone; LFD, largest follicle diameter  ^a^Fisher's exact test | | | |

| Table S2 | | | | | | |
| --- | --- | --- | --- | --- | --- | --- |
|  | **EE/GS**  **(Baseline)** | **EE/GS**  **(Final visit)** | **p-value^a^** | **DRSP**  **(Baseline)** | **DRSP**  **(Final visit)** | **p-value^a^** |
| Systolic blood pressure (mm/Hg) | | | | | | |
| - Mean±SD | 122.75±10.25 | 119.56±11.41 | 0.153 | 118.71±10.76 | 115.24±12.42 | 0.189 |
| Diastolic blood pressure (mm/Hg) | | | | | | |
| - Mean±SD | 74.31±9.49 | 72.31±12.28 | 0.261 | 73.76±8.67 | 71.06±12.22 | 0.218 |
| Weight (kg) | | | | | | |
| - Mean±SD | 58.46±10.19 | 58.68±10.53 | 0.436 | 55.78±12.47 | 55.33±12.55 | 0.137 |
| Table S2: Safety monitoring  Abbreviations: EE/GS, ethinyl estradiol 0.02 cg plus gestodene 0.075 mg; DRSP, 4 mg drospirenone; SD, standard deviation  ^a^Student t-test, | | | | | | |
